# The Association Between Cadmium Exposure and Osteoporosis: A Longitudinal Study and Predictive Model in a Chinese Female Population

**DOI:** 10.3389/fpubh.2021.762475

**Published:** 2021-11-29

**Authors:** Miaomiao Wang, Xinru Wang, Jingjing Liu, Zhongqiu Wang, Taiyi Jin, Guoying Zhu, Xiao Chen

**Affiliations:** ^1^Department of Radiology, Affiliated Hospital of Nanjing University of Chinese Medicine, Nanjing, China; ^2^Department of Occupational and Environmental Medicine, School of Public Health, Fudan University, Shanghai, China; ^3^Institute of Radiation Medicine, Fudan University, Shanghai, China

**Keywords:** cadmium, women, bone, osteoporosis, longitudinal study

## Abstract

**Objective:** The association between cadmium exposure and osteoporosis has been rarely reported in longitudinal studies. In this study, we investigated the association between osteoporosis and cadmium exposure and developed predictive models in women in a longitudinal cohort.

**Materials and Methods:** In total, 488 women living in southeastern China were included at baseline (1998). Cadmium in blood (BCd) and urine (UCd) and also renal dysfunction biomarkers and bone mineral density (BMD) were determined both at baseline and follow-up. A total of 307 subjects were finally included after excluding subjects that did not have exposure or effect biomarkers. Osteoporosis was defined based on T score ≤ −2.5. Multiple linear regression and multivariate logistic analysis were used to show the association between baseline data and follow-up osteoporosis. Based on the identified associated factors, nomograms were developed to graphically calculate the individual risk of osteoporosis.

**Results:** The baseline BMD in subjects with osteoporosis was significantly lower than that in subjects without osteoporosis (0.59 vs. 0.71 g/cm^2^, *p* < 0.05). The prevalence of low bone mass at baseline was higher in subjects with osteoporosis than in those without osteoporosis (23.5 vs. 7.2%, *p* = 0.001). Logistic regression analysis demonstrated that age [odds ratio (OR) = 1.21, 95% confidence interval (CI): 1.16–1.27], UCd (OR = 1.03, 95% CI: 1.002–1.06) and the presence of low BMD (OR = 3.84, 95% CI: 1.49–9.89) were independent risk factors for osteoporosis. For those subjects with normal baseline BMD, age, UCd, and baseline BMD were also independent risk factors for osteoporosis. The OR value was 1.16 (95% CI: 1.10–1.22) for age, 2.27 (95% CI: 1.03–4.99) for UCd > 10 μg/g creatinine, and 0.39 (95% CI: 0.21–0.72) for BMD_baseline_. We developed two nomograms to predict the risk of osteoporosis. The area under the curve was 0.88 (95% CI: 0.84–0.92) for total population and was 0.88 (95% CI: 0.84–0.92) for subjects with normal baseline BMD, respectively.

**Conclusion:** Baseline age, UCd, and BMD were associated with follow-up osteoporosis in women. Nomograms showed good performance in predicting the risk of osteoporosis.

## Introduction

Cadmium is widely distributed in the environment. In addition, some industrial activities, such as smelting and battery production, can cause environmental cadmium contamination. Cadmium can be taken up by various crops such as vegetables, rice, tobacco, and shellfish and offal (1). Cadmium in the environment can enter into the human body *via* the food chain or cigarette smoking. Bone damage is one of the severe clinical features of “Itai-Itai diseases.” Many researchers have shown that environmental exposure to cadmium may be a risk factor for bone loss or osteoporosis (2–4). Experimental studies also indicate that cadmium exposure can directly inhibit osteoblast activity or stimulate osteoclast formation (5–8). More importantly, a recent study indicated that that cadmium exposure may play a greater role in bone loss than previously thought (1). However, negative associations between cadmium levels and bone loss were also reported (9, 10). The long-term effects of cadmium on bone had substantial uncertainty (11).

Most of the population studies that focused on cadmium exposure and bone damage were of cross-sectional design. Previous studies indicated that findings of cross-sectional studies on the association between metal exposure and health effects may be limited due to the study design (12, 13). However, limited information was reported in longitudinal studies to show the relationship between cadmium exposure and bone health. Staessen et al. (14) showed that urinary cadmium was correlated with 0.01 g/cm^2^ decrease in bone density in postmenopausal women at 6.6 years of follow-up. Interestingly, a recent study investigated the association between early-life cadmium exposure and biomarkers of bone remodeling and anthropometry in 9-year-old children (15). The results showed that the cadmium level at 4.5 years of age was negatively associated with vitamin D3 levels at 9 years of age. A mild link was also found between maternal cadmium exposure and child urinary deoxypyridinoline (β = 15 nmol/L). These data indicated that cadmium exposure may be associated with future abnormal bone metabolism.

Longitudinal studies have been reported between cadmium exposure and renal function (16), child growth (17), and diabetes (18, 19). However, the association between cadmium exposure and bone mineral density (BMD) or osteoporosis has not been well studied longitudinally. Our previous study showed that the changes of BMD were associated with previous cadmium exposure level (20). However, the association between baseline cadmium exposure and the follow-up bone status was unclear. In addition, previous study mainly focused on exposure level and did not pay attention to other factors, such as baseline age and body mass index. Therefore, we conducted this study to investigate the associated factors for bone loss in a longitudinal population with cadmium exposure. Since women are more susceptible to osteoporosis, our study focused on the female population with cadmium exposure. Predictive models for prognosis or disease progress has been widely used in oncology and medicine (21). To our knowledge, only few models have been established to predict renal function in subjects with cadmium exposure (22). There is no predictive model for risk of osteoporosis in cadmium-exposed population using longitudinal data. Therefore, we also developed nomogram models to predict osteoporosis in our population.

## Materials and Methods

### Study Areas and Population

**Figure 1:**
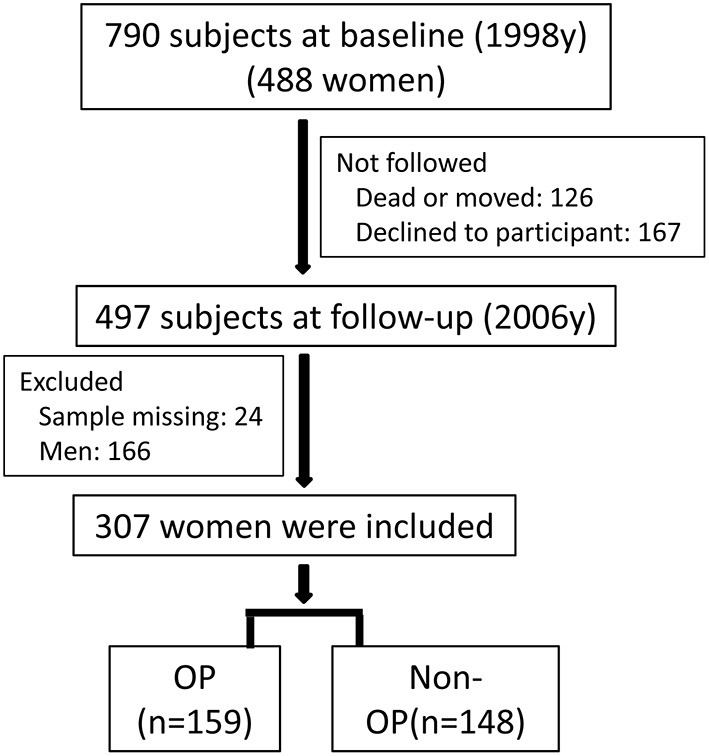
The flowchart of subjects selection. Around 790 subjects (488 women) were included at baseline (1998), and 307 women were finally included at follow-up (2006).

The study areas and population had been reported in our previous study (22, 23). In short, a survey was performed for the population living in three areas with varied levels of environmental cadmium exposure (heavy, moderate, and low exposure) in 1998. A smelt located in the southeast of China caused pollution in the heavily polluted area. The moderately polluted area and control area were 5 and 40 km away from the heavily polluted area, respectively. A total of 790 subjects living in the three areas were included (488 women and 302 men) at the baseline survey (year 1998). The subjects living in the three areas had similar lifestyle, diet habits, and social and economic status. The subjects at baseline did not have diabetes, stroke, or self-reported chronic kidney diseases. A second survey was performed in the same three areas in 2006, and 497 subjects were followed-up. Twenty-four subjects were excluded because of missing data. The present work included 307 women of 473 subjects. The flowchart of subjects selection is shown in Figure 1. A questionnaire was completed by each subject to collect demographic information, medical history, occupation, drinking and smoking habits, and menopausal status in both surveys. The Ethics Committees of Fudan University approved our surveys, and informed consent was obtained from each subject. The Declaration of Helsinki was followed during the study.

### Cadmium Analysis

Cadmium in blood (BCd) and urine (UCd) were measured using graphite-furnace atomic absorption spectrometry as described in our previous work (24). Briefly, venous blood and midstream urine were collected in cadmium-free tubes. Concentrated nitric acid was added into the samples before the determination. Quality control was also performed using cadmium reference standards. In addition, urinary *N*-acetyl-β-d-glucosaminidase (UNAG) and urinary albumin (UALB) were determined to evaluate baseline renal function. Urinary creatinine was also determined for the adjustment of UNAG, UALB, and UCd.

### Determination of Bone Mineral Density

Bone mineral density was determined at left forearm both at baseline and follow-up. At baseline, single photon absorptiometry (SPA) was used, and peripheral dual-energy X-ray absorptiometry (DXEA) (Norland, USA) was used at follow-up. Phantoms scanning before determination were performed every day for quality assurance. The T-score was used to define OP according to WHO criteria. T-score was calculated using the following equation: T-score = (X_1_ – X_2_)/SD, where X1 is the measured BMD, X_2_ is the average BMD of the same sex and young adults (30–40 years old) in control area, and SD is the standard deviation of X_2_. If the T-score was ≤ −2.5, osteoporosis was considered. Low BMD was considered if the T-score < −1.0.

### Statistical Analysis

Commercial software SPSS16.0 and R (version 3.6.4) were used for data management and statistical analysis. Qualitative data was shown as number, and quantitative data was shown as mean ± standard deviation. The Mann–Whitney U-test or two-tailed *t* tests was used to compare the data between subjects with and without osteoporosis. Multiple linear regression analysis and was used to show the association between baseline data and follow-up BMD. Multivariate logistic regression analysis was used to show the association between baseline data and follow-up osteoporosis. Based on the identified independent associated factors from multivariate logistic regression analysis (25, 26), nomograms were developed to graphically calculate the individual risk of osteoporosis. The calibration curves of the nomograms were obtained using bootstrapping with 1,000 resamples. Hosmer–Lemeshow test was used to evaluate the performance of models. Significant difference was considered when *p* was <0.05.

## Results

### The Characteristics of Subjects

A total of 307 women were included in this study, and the characteristics of the subjects are listed in Table 1. There were 159 women with OP and 148 women without OP (non-OP). The average age was higher and height was lower in OP women than these in non-OP women, respectively (*p* < 0.001). Low bone mineral density (LBMD) at baseline was significantly associated with osteoporosis (*p* = 0.001), and the mean of BMD was lower in OP subjects (*p* < 0.001). However, no significant differences were found in terms of BMI, drinking, and smoking habits. The median levels of UCd, UNAG, UALB, and BCd were higher in subjects with osteoporosis than those without osteoporosis, but no significant differences were found.

**Table 1:** The characteristic of subjects with or without osteoporosis (OP).

	**Non-OP (*n* = 148)**	**OP (*n* = 159)**	***p***
Age (years)	43.76 ± 7.14	56.72 ± 9.44	<0.01
BMD_followup_	0.75 ± 0.06	0.54 ± 0.08	<0.01
BMD_baseline_	0.71 ± 0.06	0.59 ± 0.11	<0.01
UCd (μg/g cr)	8.84 ± 11.42	10.91 ± 12.01	0.12
UNAG (U/g cr)	9.67 ± 15.72	11.43 ± 14.07	0.3
UALB (mg/g cr)	9.46 ± 13.14	10.74 ± 12.50	0.35
BCd (μg/L)	7.28 ± 9.94	8.86 ± 11.12	0.18
Height (m)	1.56 ± 0.51	1.52 ± 0.06	0
BMI (kg/m^2^)	22.94 ± 3.07	22.87 ± 3.03	0.83
Smoking	149	158	0.552
No	20	25	
Yes	129	133	
Drinking	149	158	0.123
No	149	154	
Yes	0	4	
Low BMD	149	158	0.001
No	139	128	
Yes	10	30	

*OP, osteoporosis; BMI, body index mass; BCd, cadmium in blood; UCd, urinary cadmium; cr, creatinine; NAG, N-acetyl-β-d-glucosaminidase; ALB, albumin; BMD, bone mineral density*.

### Correlation and Multiple Linear Regression Analysis

**Figure 2:**
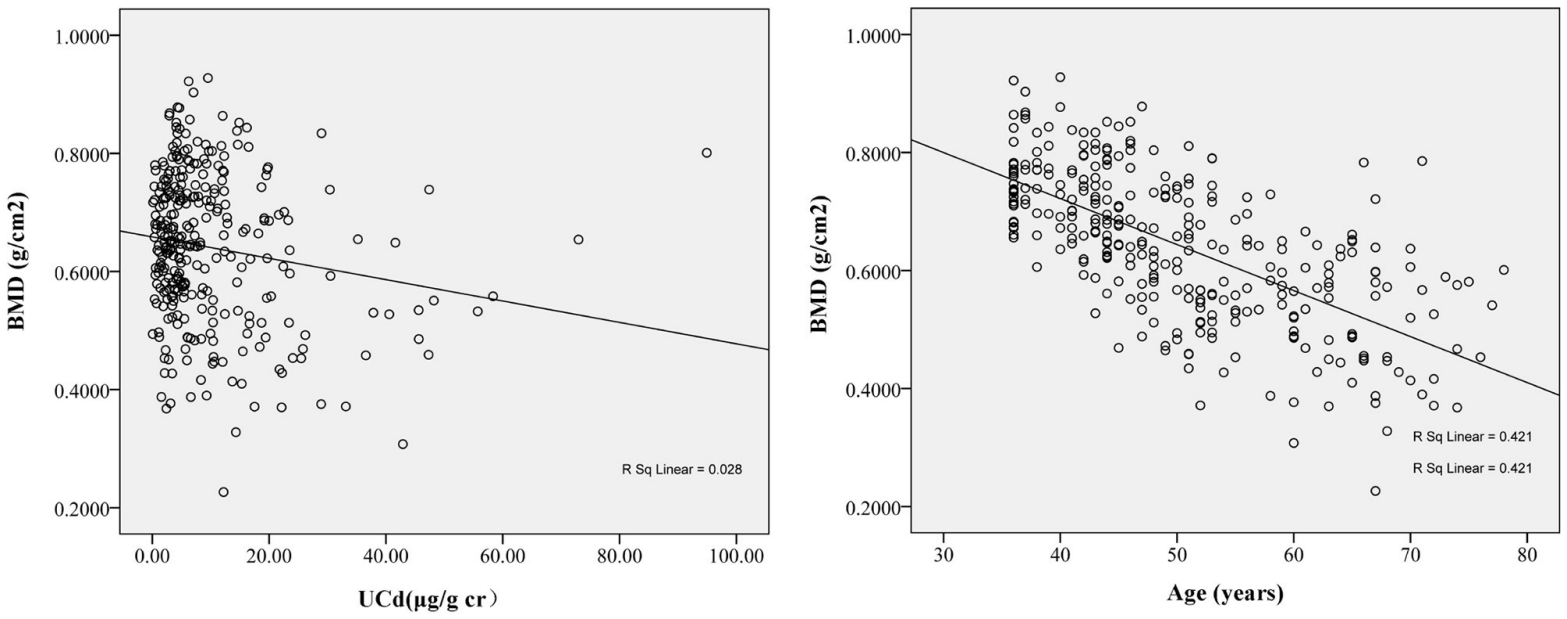
The correlation between follow-up bone mineral density (BMD) and baseline urinary cadmium (UCd) (*r* = −0.12, *p* = 0.04) and baseline age (*r* = −0.67, *p* < 0.01). The BMD at follow-up decreased with increase of baseline UCd and age. The regression equation was BMD_follow−up_= −0.003 × UCd_baseline_+0.745 for UCd and was BMD_follow−up_= −0.005 × Age_baseline_+0.992 for age.

Figure 2 shows that BMD was positively correlated with an increase in baseline UCd and age. Next, Table 2 shows the associations between markers of exposure and BMD in multiple linear regression analysis. After adjusting with UNAG, UALB, BMI, smoking, and drinking, BMD was inversely correlated with age (*p* < 0.001). In addition, BMD was positively correlated with baseline BMD and UCd level (*p* < 0.001 or *p* = 0.011).

**Table 2:** Associations between baseline variables and follow-up BMD in multiple linear regression analysis.

**Variable**	**β**	**95% CI**	***p***
Age (years)	−0.004	−0.005 to 0.002	<0.001
UCd (μg/g creatinine)	−0.002	−0.003 to 0.000	0.011
BMD (g/cm^2^)	0.599	0.476 to 0.722	<0.001
BCd (μg/L)	−0.01	−0.002 to 0.000	0.104
BMI (kg/m^2^)	0.00	−0.003 to 0.003	0.92
UNAG (U/g g creatinine)	0.00	0.000 to 0.001	0.193
UALB (mg/g creatinine)	−0.008	−0.022 to 0.006	0.250

*Model was adjusted with smoking and drinking habits*.

*BMI, body index mass; BCd, cadmium in blood; UCd, urinary cadmium; cr, creatinine; NAG, N-acetyl-β-d-glucosaminidase; ALB, albumin; BMD, bone mineral density*.

### Multiple Logistic Regression

As shown in Table 3, multivariable logistic regression analysis was used to identify the associated factors for osteoporosis. The odds ratio (OR) value was 1.20 (95% CI: 1.15–1.26) for age, 1.03 (95% CI: 1.001–1.05) for UCd, and 3.71 (95% CI: 1.45–9.54) for baseline low BMD. Similar results were also observed if adjusting for smoking, drinking, menopausal status, BMI, and renal function (OR = 1.21, 95% CI: 1.16–1.27; OR = 1.03, 95% CI: 1.002–1.06; OR = 3.84, 95% CI: 1.49–9.89). If BCd was defined as exposure biomarker, the OR value was 1.21 (95% CI: 1.16–1.27) for age, 1.03 (95% CI: 0.99–1.06) for BCd, and 3.12 (95% CI: 1.22–7.80) for baseline low BMD after adjusting for possible confounders.

**Table 3:** Adjusted ORs (95% CI) of incident of osteoporosis in total population and women with normal baseline BMD.

**Populations**	**Variables**	**Model 1**	**Model 2**	**Model 3**
Total population	Age (y)	1.20 (1.15, 1.26)	1.21 (1.16, 1.26)	1.21 (1.16, 1.27)
	UCd (μg/g creatinine)	1.03 (1.001, 1.05)	1.03 (1.001, 1.05)	1.03 (1.002, 1.06)
	LBMD (yes vs. no)	3.71 (1.45, 9.54)	3.84 (1.49, 9.87)	3.84 (1.49, 9.89)
Total population	Age	1.20 (1.15, 1.26)	1.21 (1.16, 1.27)	1.21 (1.16, 1.27)
	BCd (μg/L)	1.02 (0.99, 1.06)	1.03 (1.00, 1.06)	1.03 (0.99, 1.06)
	LBMD (yes vs. no)	3.12 (1.21, 8.05)	3.12 (1.22, 7.99)	3.12 (1.22, 7.80)
Women with normal baseline BMD	Age (y)	1.15 (1.09, 1.21)	1.16 (1.10, 1.22)	1.16 (1.10, 1.22)
	UCd (μg/g cr)			
	0–5	1	1	1
	5–10	1.53 (0.67,3.50)	1.43 (0.62, 3.30)	1.45 (0.54,2.50)
	>10	2.24 (1.06,4.74)	2.18 (1.03, 4.65)	2.27 (1.03,4.99)
	BMD ( ×10, g/cm^2^)	0.35 (0.19,0.65)	0.39 (0.21, 0.72)	0.39 (0.21,0.72)

*Model 2 was adjusted with smoking, drinking, menopausal status and body mass index*.

*Model 3 was additionally adjusted with urinary N-acetyl-β-d-glucosaminidase and urinary albumin*.

*BCd, cadmium in blood; UCd, urinary cadmium; cr, creatinine; BMD, bone mineral density; LBMD, low BMD; OR, odds ratio*.

Subsequently, we divided UCd into three groups (<5.0, 5–10, and > 10.0 μg/L), and identified the risk factors for osteoporosis in women with normal baseline BMD (Table 3). The OR value was 1.15 (95% CI: 1.09–1.21) for age, 2.24 (95% CI: 1.06–4.74) for UCd > 10 μg/L, and 0.35 (95% CI: 0.19–0.65) for baseline BMD. Approximate values were found after adjusting with confounders.

### Nomogram Model

**Figure 3:**
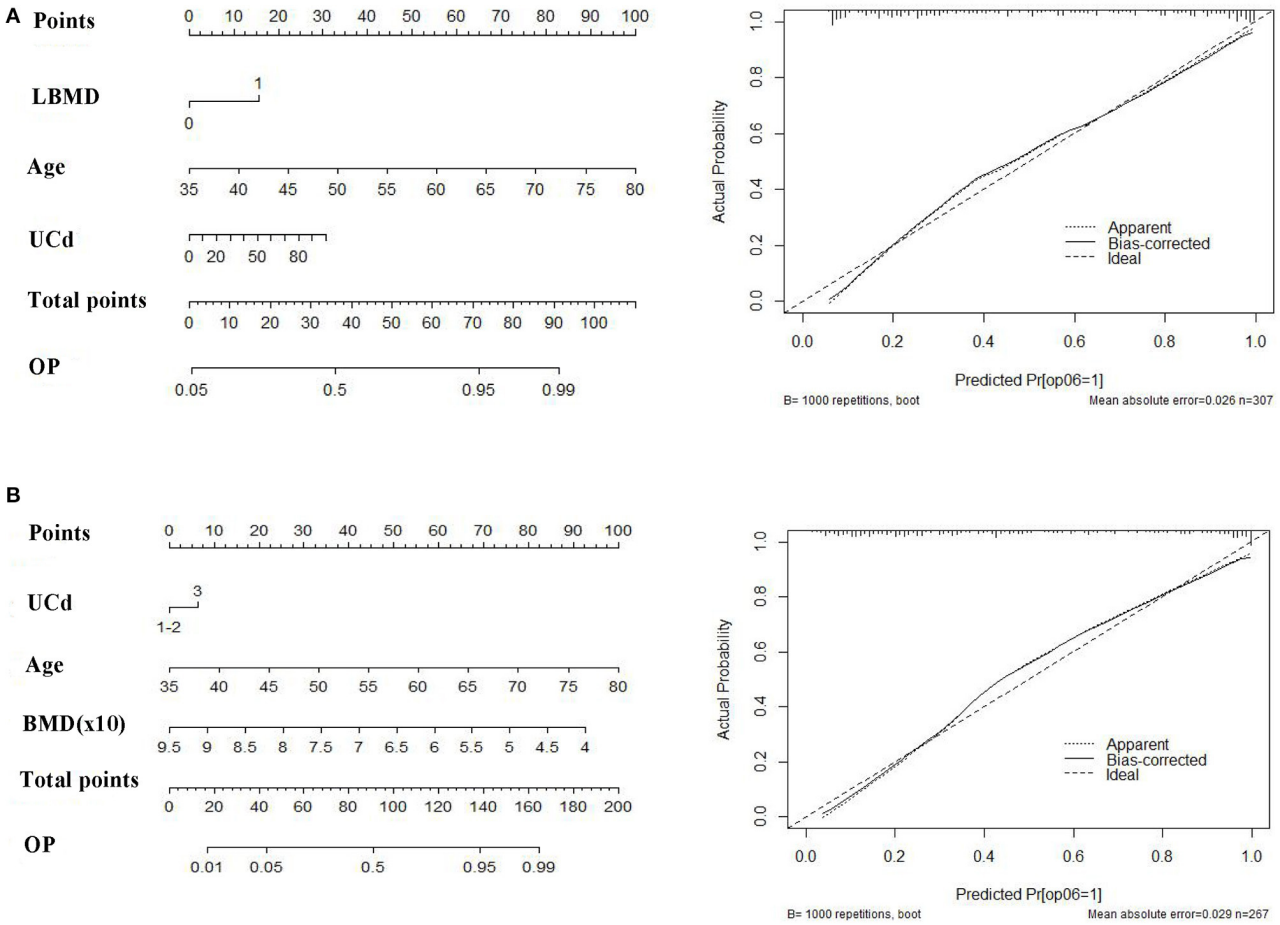
The nomograms (right) and calibration curves (left) to predict follow-up osteoporosis in all population **(A)** and women with normal baseline bone mineral density (BMD) **(B)**. Age, UCd (quantitative data) and presence of low BMD (0, no; 1, yes) were included for total population. Age, UCd (qualitative data) and baseline BMD (quantitative data) were included for women with normal baseline BMD. UCd was divided into three groups: 0–5 (1), 5–10 (2), and >10 μg/g creatinine (3).

Based on the associated factors obtained from logistic regression analyses, we developed two nomograms to predict the risk of osteoporosis. For all women, age, UCd, and low BMD were included in the nomogram (Figure 3A). Age, UCd, and BMD were included in the nomogram for subjects with normal BMD at baseline (Figure 3B). Based on these factors, we obtained the sum of each score (total points), which could individually predict the risk. The calibration curves of the nomograms for the probability of osteoporosis using bootstrapping with 1,000 resamples demonstrated good agreement between prediction and observation. Subsequently, we evaluated the performance of the above two nomograms in predicting the probability of osteoporosis. The area under the curve (AUC) was 0.88 (95%CI: 0.84–0.92) with a sensitivity of 89.2% (95% CI: 83.3–93.6%) and a specificity of 68.5% (60.3–75.8%) for all women and it was 0.88% (95% CI: 0.84–0.92) with a sensitivity of 77.3% (95% CI: 69.1–84.3%), and a specificity of 86.3% (79.5–91.6%) for subjects with normal baseline BMD, respectively. Hosmer–Lemeshow test also showed good performance (*p* = 0.75 and *p* = 0.12, respectively).

## Discussion

Cadmium exposure may be one of the risk factors for osteoporosis and bone fractures. However, most of the studies to date were of cross-sectional design. The role of cadmium exposure on bone health has not been well recognized in longitudinal studies. Based on China Cd study that was performed in 1998 (24), a follow-up survey was conducted in 2006 (10). We identified the associated factors for follow-up BMD/osteoporosis. Our results showed that baseline UCd levels, age, and BMD were significantly associated with follow-up BMD, and risk of osteoporosis. Interestingly, for those subjects with a normal baseline BMD, a significant association was also found between UCd levels and follow-up osteoporosis. Moreover, based on the independent associated factors for follow-up osteoporosis, we developed two nomogram models to predict future osteoporosis which showed high predictive performance.

Urinary Cadmium is a biomarker of past and lifetime cadmium exposure (2). Many cross-sectional studies have shown the association between UCd levels and bone mineral density or risk of osteoporosis (10, 27, 28). Several researchers did not observe statistical differences among the quartiles of UCd concentrations and BMD or osteoporosis (9, 10, 29). Our previous study showed that UCd was associated with BMD changes (20) during the follow-up. In the present study, our data further showed that UCd was related to bone loss or osteoporosis. Similar results were also reported in a prospective study with a 6.6 years follow-up (14). However, no strong associations were found between BCd levels and follow-up BMD or osteoporosis. This may be due to the fact that BCd mainly reflected recent exposure. We also performed a subgroup analysis on subjects with normal baseline BMD. UCd > 10 μg/g cr showed a 2.24-fold of osteoporosis compared with those with low levels of UCd (less than 5 μg/g cr). This association remained significant after adjusting for confounders. These data further indicated that cadmium exposure was associated with bone loss.

Baseline age and BMD or the presence of low BMD were another two independent risk factors for osteoporosis in our study. The incidence of osteoporosis was increased with age both in men and women (30). It is also easy to understand that baseline BMD is one of determination of the future risk of osteoporosis. Increasing age and lower bone mineral density or low T score were also related to bone fractures (31, 32).

Predictive models such as nomograms have been widely used in clinical studies to predict prognosis, therapeutic effects, death, or recurrence (21, 33). A few studies also adopted nomograms to predict risk of bone fractures for postmenopausal women or men (32, 34). A nomogram was also used to predict cadmium-related bone loss or fractures in a cross-sectional cohort (22). Studies with longitudinal design may be more suitable for such a prediction model. Based on those risk factors, we developed two nomograms to predict risk of osteoporosis. Those models showed good performance as shown by high AUC values. The nomograms may have potential in management of bone status in a population with cadmium exposure. In addition, from the nomograms we can see that baseline age had a great contribution to risk of osteoporosis. For example, the score was 35 for a 50 year woman, 8 for a woman with UCd of 20 μg /g cr, and was 15 for a person with baseline low BMD. These data may indicate that the contribution of cadmium exposure to bone loss was not so great, even though significant association was found between UCd levels and osteoporosis.

Our study had strengths and limitations. One major strength was the longitudinal design with a 8-year follow-up. Another strength was that we developed predictive model using nomograms which can visually, individually. and quantitatively show the risk. In addition, we also performed subgroup analysis in women with normal baseline BMD which may give a strong association between cadmium exposure and osteoporosis. Limitations included: (1) the sample sizes are not large; (2) BMD was not obtained from central axial bone; (3) the association between bone fracture and cadmium exposure was not investigated; (4) only female population was included in our study; (5) there was no external validation because such follow-up study in cadmium-exposed population is seldom. However, we performed subgroup analysis which could be considered as a validation.

In conclusion, our study showed that cadmium exposure was associated with BMD and osteoporosis in women in a longitudinal cohort. Age and baseline BMD were also risk factors for osteoporosis. Moreover, we developed two nomograms to predict the individualized risk of osteoporosis, which could help effectively manage osteoporosis in subjects with cadmium exposure. Further studies are required to externally validate the nomograms.

## Data Availability Statement

The original contributions presented in the study are included in the article/supplementary material, further inquiries can be directed to the corresponding author/s.

## Ethics Statement

The studies involving human participants were reviewed and approved by Ethics Committee of Fudan University. The patients/participants provided their written informed consent to participate in this study.

## Author Contributions

TJ, GZ, and XC obtained the data, conceptualized the manuscript, participated in study design, and reviewed the draft of manuscript. MW, XW, and JL analyzed the data and wrote the first draft of manuscript. ZW assisted with the initial data analyses and review the draft of manuscript. All the authors have read and approved the manuscript.

## Funding

This study was funded by National Natural Science Foundation of China (Nos. 81773460, 81102148), Educational Commission of Jiangsu Province (KYCX21_1632) and the European Union (PHIME, FOOD-CT-2006-016253).

## Author Disclaimer

The article reflects only the authors' views, and the European Union is not liable for any use that may be made of the information.

## Conflict of Interest

The authors declare that the research was conducted in the absence of any commercial or financial relationships that could be construed as a potential conflict of interest.

## Publisher's Note

All claims expressed in this article are solely those of the authors and do not necessarily represent those of their affiliated organizations, or those of the publisher, the editors and the reviewers. Any product that may be evaluated in this article, or claim that may be made by its manufacturer, is not guaranteed or endorsed by the publisher.
